# Timing of postoperative chemotherapy and prognosis in neoadjuvant-treated gastric cancer patients: a multicenter real-world cohort study

**DOI:** 10.1080/07853890.2025.2500690

**Published:** 2025-05-07

**Authors:** Hua-Long Zheng, Ling-Kang Zhang, Hong-Hong Zheng, Chen-Bin Lv, Bin-Bin Xu, Guang-Tan Lin, Qi-Yue Chen, Jian-Xian Lin, Chao-Hui Zheng, Chang-Ming Huang, Jian-Wei Xie

**Affiliations:** ^a^ Department of Gastric Surgery, Fujian Medical University Union Hospital, Fuzhou, Fujian, China; ^b^ Fujian Provincial Minimally Invasive Medical Center, Fuzhou, Fujian, China; ^c^ Department of General Surgery, Fujian Medical University Union Hospital, Fuzhou, Fujian, China; ^d^ Department of Gastrointestinal Surgery, Zhangzhou Affiliated Hospital of Fujian Medical University, Zhangzhou, Fujian, China; ^e^ Department of Digestive Endoscopy, Fuzhou University Affiliated Provincial Hospital, Fujian Provincial Hospital, Fuzhou, Fujian, China

**Keywords:** Locally advanced gastric cancer, neoadjuvant chemotherapy, adjuvant chemotherapy, time to chemotherapy

## Abstract

**Background:**

The optimal time to chemotherapy (TTC) in locally advanced gastric cancer (LAGC) patients treated with neoadjuvant chemotherapy (NLAGC) remains unclear.

**Methods:**

Consecutive 524 patients with NLAGC between Jan. 2010 and Dec. 2022 were identified. Patients were categorized into three groups: TTC < 6w, 6w ≤ TTC ≤ 8w, and TTC > 8w. Survival analysis was conducted using the Cox proportional hazards model to assess the impact of TTC on gastric cancer-specific mortality (GCSM) and all-cause mortality (ACM). Cumulative competing risk curves were employed to evaluate the incidence of competing events.

**Results:**

Overall, 451 patients were included.Cumulative competing risk curves showed that the 3-year ACM and GCSM were significantly lower in the 6w ≤ TTC ≤ 8w group (ACM: 19.7% vs. 37.2% vs. 39.7%, GCSM: 19.7% vs. 35.2% vs. 38.8%) compared to the TTC < 6w and TTC > 8w groups. Compared to patients with 6w ≤ TTC ≤ 8w, those with TTC < 6w or >8w had an increased risk of GCSM (HR: 2.792 and HR: 2.343, respectively) and ACM (HR: 3.102 and HR: 2.719, respectively) after adjusting for confounders. Furthermore, 6w ≤ TTC ≤ 8w had later peak recurrence compared to TTC < 6w and TTC > 8w (Peak months: 9.7 *vs.* 4.3 *vs.* 3.1).

**Conclusion:**

A postoperative chemotherapy timing of 6–8 weeks was associated with better survival and delayed recurrence in NLAGC patients. These findings suggest that the 6–8 week time-window should be a key timeframe for personalized postoperative adjuvant chemotherapy decisions.

## Introduction

In 2020, more than 1 million new cases of gastric cancer (GC) were diagnosed globally, with ∼783,000 GC-related deaths, rendering it the fifth most common and fourth most lethal cancer worldwide [1,2]. Surgical resection remains the cornerstone of treatment for resectable gastric cancer; however, its efficacy as a monotherapy is limited [3]. The MAGIC trial in 2006 [4] was pivotal in demonstrating that perioperative chemotherapy combined with surgery significantly improved overall survival and progression-free survival compared to surgery alone. Similarly, in 2011, a phase III clinical trial conducted by Ychou et al. produced analogous findings [5]. Khrizman et al. [6] also ­highlighted that even in specialized cancer centers, a considerable proportion of patients undergoing neoadjuvant treatment with curative intent do not complete postoperative chemotherapy, underscoring the need for strategies to promote completion of this third and final stage of treatment following neoadjuvant chemotherapy and surgery. Consequently, the integration of perioperative chemotherapy with surgery has emerged as a novel therapeutic approach for Locally Advanced Gastric Cancer (LAGC) [7–10].

Previous studies have suggested that prolonged intervals between surgery and adjuvant chemotherapy may increase the risk of micrometastatic expansion. Delayed chemotherapy could hinder the early inhibition of angiogenesis in micrometastases and contribute to the development of initial drug resistance [11,12]. Additionally, animal models have shown that surgery may lead to an increase in circulating tumor cells, which could promote metastatic growth [13,14]. This process has been linked to reduced angiogenesis and elevated tumorigenic growth factors. Based on these findings, it has been proposed that excessive delays in adjuvant chemotherapy may negatively impact treatment outcomes. Previous studies on breast, colorectal, and pancreatic cancers have demonstrated a close association between the timing of adjuvant chemotherapy initiation and tumor prognosis [15–21]. Initiating postoperative chemotherapy within the appropriate time window can help reduce recurrence and improve survival rates. For instance, a meta-analysis revealed that patients with stage III colorectal cancer (CRC) should commence adjuvant chemotherapy within eight weeks postoperatively because initiating chemotherapy beyond this timeframe significantly diminishes overall survival [15]. Moreover, Gagliato et al. observed that in specific subsets of patients with breast cancer (those with stage III TNM, TNBC, and Her2-positive tumors), delayed chemotherapy [time to chemotherapy (TTC) > 61 days] was associated with poor prognosis [16]. Hence, the prompt administration of chemotherapy is recommended for high-risk patients. Furthermore, many researchers have focused on the impact of the initiation time of postoperative adjuvant chemotherapy on the prognosis of patients with directly operable gastric cancer [22–24]. Studies have found that delaying TTC beyond six weeks is significantly associated with an increased risk of local recurrence and mortality [25]. Huang et al. indicated that patients who commenced chemotherapy more than 8 weeks after surgery exhibited poorer long-term prognosis than those with TTC < 8 weeks (with respective 5-year overall survival rates of 56.6 and 40.2%, and corresponding 5-year recurrence-free survival rates of 57.6 and 46.4%) [26].

In recent years, significant progress has been made in neoadjuvant chemotherapy for locally advanced gastric cancer (LAGC), particularly in combination treatment strategies. For instance, a Phase II trial by Zhao et al. [27] demonstrated that camrelizumab (a PD-1 inhibitor) combined with mFOLFOX achieved a 9.1% pathological complete response (pCR) rate, with HER2 and CTNNB1 mutations potentially associated with treatment sensitivity. Additionally, multi-drug regimens, such as docetaxel, oxaliplatin, and S-1 (DOS), have shown promising efficacy in the JCOG1704 Phase II trial [28] and the Neo-REGATTA study [29], suggesting that successful neoadjuvant chemotherapy followed by radical surgery may improve patient outcomes. However, despite advances in perioperative chemotherapy regimens and the evolving role of neoadjuvant therapy, key postoperative treatment questions remain unresolved, including TTC and the ideal duration of adjuvant chemotherapy, both of which require further investigation.

With the widespread adoption of perioperative chemotherapy, many researchers have explored the TTC postoperatively in patients undergoing neoadjuvant chemotherapy (NACT) followed by curative surgery. Research indicates that irrespective of cancer type (breast, colorectal, or pancreatic) and NACT administration, the timing of chemotherapy initiation post-surgery significantly correlates with patient survival prognosis. Thong et al. found that CRC patients in the non-delayed group at 23 (proposed) and 25 (median) weeks’ cutoff reported better 5-year disease-free survival (DFS) compared to those in the delayed group by 4.1 and 0.8%, respectively. Conversely, at a cutoff of 28 (mean) weeks, the delayed group exhibited better DFS by 4.4% [30]. After investigating the time to initiation of postoperative radiotherapy (PORT) in breast cancer patients receiving neoadjuvant chemotherapy, Saulo et al. discovered that receiving PORT at 8 weeks postoperatively was associated with superior disease-free survival (DFS) (<8 *vs.* 8–16 weeks: HR 0.33; 95% CI 0.13–0.81; *p* = 0.02; <8 *vs.* >16 weeks: HR 0.38; 95% CI 0.15–0.96; *p* = 0.04) and overall survival (OS) (<8 *vs.* 8–16 weeks: HR 0.22; 95% CI 0.05–0.90; *p* = 0.036; <8 *vs.* >16 weeks: HR 0.28; 95% CI 0.07–1.15; *p* = 0.08). They concluded that early initiation of PORT is imperative [31].

With the advancements in perioperative treatment for gastric cancer, there has been a steady rise in the number of patients with LAGC undergoing NACT. These patients can benefit from multidisciplinary treatment approaches, including chemotherapy, radiotherapy, and targeted therapy, which have the potential to improve prognosis and enhance the success rate of surgical resection and long-term survival [32,33]. However, there is currently a paucity of research exploring the optimal timing for initiating postoperative chemotherapy in patients with LAGC who have undergone NACT followed by gastrectomy. Thus, this study aims to utilize real-world multicenter data to elucidate the impact of various postoperative chemotherapy initiation times on the prognosis of patients with NLAGC. The objective of this study is to provide clinical practitioners with valuable insights to aid in treatment decision-making.

## Materials and methods

### Patient population

**Figure 1.**
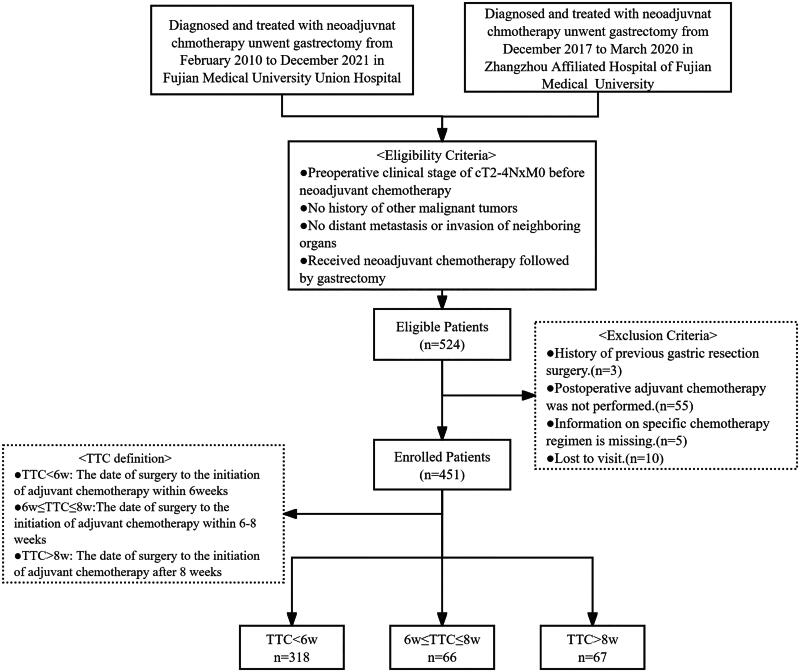
Flow chart of the charge and arrangement standard.

This retrospective study analyzed the clinicopathological data of 524 LAGC patients who underwent D2 radical gastrectomy following NACT between January 2010 and December 2021 at two centers: Fujian Medical University Union Hospital (FJMUUH) and Zhangzhou Affiliated Hospital of Fujian Medical University (ZAHFMU). Patients were selected based on the following criteria: (1) Diagnosis of locally advanced gastric cancer with a clinical stage of cT2–T4, N×, M0 before neoadjuvant chemotherapy; (2) No coexisting or previous malignancies; (3) Absence of distant metastasis or direct invasion into adjacent organs confirmed by imaging and clinical evaluation; (4) Completion of gastrectomy following neoadjuvant chemotherapy. Exclusion criteria included: (1) Previous history of gastric resection; (2) Recent (<3 months) cardiovascular events, including cerebrovascular or coronary artery disease; (3) Undergoing emergency surgical intervention; (4) Failure to receive adjuvant chemotherapy postoperatively; (5) Incomplete data on chemotherapy regimen; (6) Inadequate follow-up information or loss to follow-up. The relevant inclusion and exclusion criteria have been described in our previous studies conducted [34,35]. After applying the exclusion criteria, 451 patients were included in this study. A flowchart depicting this process is shown in Figure 1.

Due to the long study period, chemotherapy regimens evolved with advancements in clinical guidelines and the needs for individualized treatment. Supplemental Figure 5 presents a comparison of chemotherapy regimen differences across different time periods, and the results show that: 2010–2013y: Platinum-based regimens (78.8%) (e.g. ECF and XELOX) were predominant. 2014–2017y: While platinum-based regimens (62.0%) remained widely used, the proportion of paclitaxel-based regimens (3.8→15.5%) and other regimens (17.3→22.5%) gradually increased. 2018–2022y: The proportion of paclitaxel-based regimens and other regimens further increased (21.1→38→71%).

This study was conducted in accordance with the ethical principles outlined in the World Medical Association’s Declaration of Helsinki. Informed consent was obtained from all patients and their legal guardians. The study protocol was approved by the Ethics Board of both hospitals (2024KY039), and written informed consent was obtained from all participants.

### Treatments

In this study, all patients received neoadjuvant chemotherapy (NACT) centered on fluorouracil-based regimens. The most commonly combined agents included platinum compounds (cisplatin, oxaliplatin, or loplatin) and taxanes (paclitaxel or docetaxel). Postoperative adjuvant chemotherapy (AC) was routinely recommended, comprising three general categories: (1) platinum-based regimens (e.g. platinum plus capecitabine, S-1, or 5-FU); (2) taxane-based combinations (e.g. paclitaxel with capecitabine, S-1, or 5-FU); (3) other regimens, such as paclitaxel-platinum or single-agent S-1. Standardized regimens included SOX/XELOX protocols, administered as two preoperative and six postoperative cycles at 3-week intervals. In these regimens, S-1 (40–60 mg/m^2^) or capecitabine (1000 mg/m^2^, twice daily, days 1–14) was combined with oxaliplatin (130 mg/m^2^, day 1). Alternatively, FOLFOX4 was used in three preoperative and six postoperative cycles every 2 weeks, consisting of oxaliplatin (85 mg/m^2^, day 1), folinic acid (200 mg/m^2^, 2-h infusion), followed by a bolus of fluorouracil (400 mg/m^2^) and a 22-h continuous infusion of fluorouracil (600 mg/m^2^). The chemotherapy regimens and doses aforementioned have been previously documented [34,36]. Paclitaxel regimens, platinum-based regimens, and other regimens were evenly distributed across the entire patient cohort (39.9 *vs.* 39.9 *vs.* 20.2%) (see Supplemental Figure 1A). All perioperative chemotherapy regimens and doses were tailored based on tumor response and chemotherapy toxicity.

The surgical procedure entailed gastrectomy with D2 lymph node dissection, the extent of which was determined in accordance with the *Japanese gastric cancer treatment guidelines 2021 (version 6th)* [7]. The R status was evaluated through intraoperative frozen section analysis of surgical margins and postoperative pathological reports [34].

### Data collection

Clinical, pathological, and follow-up information were retrospectively extracted from the Gastric Cancer Databases maintained by two tertiary medical centers in China. The dataset included demographic parameters, such as sex, age at diagnosis, body mass index (BMI), and American Society of Anesthesiologists (ASA) physical status classification. Tumor-related and perioperative variables were also collected, including tumor size and location, pathological ypTNM stage, resection margin status (R0/R1), presence of lymphovascular and perineural invasion, surgical duration, intraoperative blood loss, occurrence of postoperative complications, and hospital length of stay. In addition, we retrieved treatment-specific data, such as the number and regimen of neoadjuvant chemotherapy (NACT) cycles, whether postoperative adjuvant chemotherapy (AC) was initiated, total AC cycles, and the corresponding regimens administered. These variables were selected based on prior studies linking them to long-term outcomes in patients undergoing perioperative treatment for gastric cancer.

### Definition and patient groups

TTC was defined as the interval from the date of surgery to the initiation of adjuvant chemotherapy (see Supplemental Figure 2). According to the 6th edition of the Japanese Gastric Cancer Treatment Guidelines (updated in 2021) [7], it is clearly recommended that postoperative chemotherapy should be initiated within 6 weeks to achieve optimal efficacy. Additionally, several high-quality studies [15,18] have shown that starting chemotherapy within 8 weeks postoperatively significantly improves survival rates, while starting chemotherapy after 8 weeks is associated with poorer prognosis. These studies provide scientific evidence for using 8 weeks as the upper boundary. Furthermore, studies on other cancers [16] have also indicated that the 6–8 week period is a critical timeframe for initiating chemotherapy. Thus, based on the TTC, patients were divided into three groups: within 6 weeks (TTC < 6w), within 6–8 weeks (6w ≤ TTC ≤ 8w), and after 8 weeks (TTC > 8w).

Primary outcomes were GCSM (death due to gastric cancer, with censoring of other causes of death and survivors) and all-cause mortality (ACM) (death from any cause, with censoring of survivors). Recurrence-free survival (RFS) was defined as the time from the end of surgery to the first recorded instance of cancer recurrence, metastasis, or occurrence of a new cancer.

The tumor regression grade (TRG), representing the histopathological response to neoadjuvant chemotherapy, was evaluated based on the criteria outlined in the 8th edition of the AJCC Cancer Staging Manual. Similarly, the post-treatment T (tumor) and N (lymph node) stages were determined according to the AJCC 8th edition TNM classification system [37].

### Follow-up

Postoperative follow-up was conducted at regular intervals: every three months during the first two years and biannually thereafter until five years post-surgery. The routine surveillance protocol included periodic physical assessments, hematologic testing, and cross-sectional imaging (chest X-ray and abdominal CT scans) every six months for the initial three years. In addition, annual upper gastrointestinal endoscopy was performed for up to three years postoperatively. PET/CT was selectively employed in cases where tumor recurrence was clinically suspected [35,36,38].

### Statistical analysis

Correlation analysis was performed using SPSS version 25 (IBM, Armonk, NY, USA) and R version 4.3.3 (http://www.r-project.org). Continuous variables were expressed as mean ± standard deviation (SD) if normally distributed; otherwise, they were represented as median (interquartile range, IQR). The chi-square test or Fisher’s exact test was used to analyze categorical variables, presented as percentages. Kernel density curves assessed the peak recurrence time across different TTC groups. The Cox proportional hazards model was employed to identify independent risk factors associated with the patient’s long-term prognosis, with all variables from the univariate analysis with *p* < 0.05 included in the multivariate analysis. Competing risk models (Fine and Gray) were employed to account for the potential impact of competing events, such as recurrence and death from other causes, on survival outcomes. This model was used to assess the cumulative incidence of gastric cancer-specific mortality (GCSM) and all-cause mortality (ACM) while adjusting for key confounding factors. By incorporating this approach, we ensured that the influence of competing events on the relationship between chemotherapy timing and survival outcomes was appropriately controlled [39]. Cox regression analysis assessed hazard ratios (HRs) and 95% confidence intervals (CIs) for ACM and GCSM.

## Results

### Baseline characteristics

Table 1 presents the baseline characteristics of three groups of patients: TTC < 6 weeks, 6w ≤ TTC ≤ 8w, and TTC > 8 weeks, with 451 patients included. TTC < 6w comprises 318 cases (70.5%), while 66 cases (14.7%) fall within the range of 6w ≤ TTC ≤ 8w, and 67 cases (14.8%) exceed TTC > 8w. In the comparison of baseline data across the three groups, statistically significant differences were observed only in age and postoperative AC Cycles. No significant statistical differences were observed in Sex, BMI, ASA classification, ypTNM stage, tumor location, tumor size, TRG grade, lymphovascular invasion, neural invasion, R status, number of neoadjuvant chemotherapy cycles. In addition, we found that there were no significant statistical differences among the groups regarding postoperative recovery: the results showed no notable differences in postoperative hospital stay, postoperative complications (yes or no), or Clavien–Dindo classification (All *p* > 0.05).

**Table 1.** Characteristic baseline.

Characteristic	TTCs			p-Value^a^
	TTC < 6w *N* = 318, *n* (%)	6w ≤ TTC ≤ 8w *N* = 66, *n* (%)	TTC > 8w *N* = 67, *n* (%)	
Age, n (%)				**0.019**
<65	190 (59.7)	27 (40.9)	38 (56.7)	
≥65	128 (40.3)	39 (59.1)	29 (43.3)	
Sex, n (%)				0.093
Male	234 (73.6)	50 (75.8)	41 (61.2)	
Female	84 (26.4)	16 (24.2)	26 (38.8)	
BMI, n (%)				0.093
<25 kg/m^2^	263 (82.7)	47 (71.2)	55 (82.1)	
≥25 kg/m^2^	55 (17.3)	19 (28.8)	12 (17.9)	
ASA, n (%)				0.815
1	46 (14.5)	7 (10.6)	10 (14.9)	
2	227 (71.4)	50 (75.8)	45 (67.2)	
3	45 (14.2)	9 (13.6)	12 (17.9)	
ypTNM stage, n (%)				0.157
ypCR/I	60 (18.9)	17 (25.8)	12 (17.9)	
II	106 (33.3)	20 (30.3)	14 (20.9)	
III	152 (47.8)	29 (43.9)	41 (61.2)	
Location, n (%)				0.896
Upper	137 (43.1)	30 (45.5)	27 (40.3)	
Middle	62 (19.5)	13 (19.7)	11 (16.4)	
Lower	93 (29.2)	20 (30.3)	22 (32.8)	
Mixed	26 (8.2)	3 (4.5)	7 (10.4)	
Tumor size, n (%)				0.520
<5 cm	187 (58.8)	35 (53)	42 (62.7)	
≥5 cm	131 (41.2)	31 (47)	25 (37.3)	
TRG, n (%)				0.352
0/1	76 (23.9)	21 (31.8)	15 (22.4)	
2/3	242 (76.1)	45 (68.2)	52 (77.6)	
Lymphovascular invasion, n (%)				0.567
No	191 (60.1)	35 (53)	40 (59.7)	
Yes	127 (39.9)	31 (47)	27 (40.3)	
Neural invasion, n (%)				0.248
No	160 (50.3)	37 (56.1)	28 (41.8)	
Yes	158 (49.7)	29 (43.9)	28 (41.8)	
R status, *n* (%)				0.292
R0	286 (89.9)	56 (84.8)	57 (85.1)	
R1	32 (10.1)	10 (15.2)	10 (14.9)	
NACT cycles, median (IQR)	4 (3–4)	4 (3–4)	3 (3–4)	0.213
AC cycles, median (IQR)	4 (2–6)	4 (3–6)	3 (1–4)	**<0.001**
AC, n (%)				**0.019**
<4 cycles	113 (35.5)	24 (36.4)	36 (53.7)	
≥4 cycles	205 (64.5)	42 (63.6)	31 (46.3)	
Surgical outcomes				
Blood loss, median (IQR)	50 (30–50)	50 (30–60)	50 (30–65)	0.426
The length of operation, median (IQR)	184 (165–210)	190 (160–230)	180 (152–232)	0.472
Postoperative hospital stay, median (IQR)	9 (7–12)	8 (7–10)	8 (7–12)	0.054
Complications, n (%)				0.132
No	254 (79.9)	46 (69.7)	49 (73.1)	
Yes	64 (20.1)	20 (30.3)	18 (26.9)	
Clavien-Dindo				0.421
I	44 (68.8)	15 (75)	10 (55.6)	
≥II	20 (31.2)	5 (25)	8 (44.4)	

BMI: body mass index; ASA: American Society of Anesthesiologists; ypCR/I, II, III: pathological nodal stage after neoadjuvant chemotherapy; tumor size: maximum diameter of the tumor; location: location of the tumor; TRG: tumor regression grade.

Bold values indicated that the *p*-value <0.05.

^a^ One-way ANOVA; Pearson’s chi-squared test; Fisher’s exact test.

In the different TTC subgroups, the distribution of chemotherapy regimens showed relatively consistent patterns [6w ≤ TTC ≤ 8w: Platinum-based regimens: 26 (33.3%); Paclitaxel regimens: 29 (37.2%); Other regimens: 11 (14.1%). TTC < 6w: Platinum-based regimens: 121 (38.4%); Paclitaxel regimens: 129 (41.0%); Other regimens: 68 (21.6%). TTC > 8w: Platinum-based regimens: 33 (46.5%); Paclitaxel regimens: 22 (31.0%); Other regimens: 12 (16.9%)] (see Supplemental Figure 1B). In terms of chemotherapy cycles, 4 cycles dominated both neoadjuvant and postoperative adjuvant chemotherapy, accounting for 40.1 and 22.8%, respectively. Neoadjuvant chemotherapy patients were more concentrated in 3–4 cycles, while postoperative adjuvant chemotherapy showed a more even distribution, with relatively more patients concentrated in 4 cycles. Overall, 4 cycles of chemotherapy were the most common in both treatment phases (see Supplemental Figure 3).

Supplemental Table 1 shows the intergroup distribution of variables across different chemotherapy regimens and chemotherapy initiation times (TTC < 6w, 6w ≤ TTC ≤ 8w, TTC > 8w). The results indicated that the overall distribution of variables was relatively balanced between the groups, with most variables showing no significant differences in *p*-values across the groups. Patients with different chemotherapy regimens and initiation times were generally similar in baseline characteristics. Additionally, except for the *p*-value for age in the Other regimens group (0.04), no statistically significant differences were observed in the variables for the Platinum-based regimens and Paclitaxel regimens groups, indicating a generally homogeneous distribution of characteristics between the groups.

There were no significant differences in the incidence of chemotherapy-related adverse events among the different TTC groups (*p* = 0.978). The most commonly observed adverse events across all groups included leukopenia, hypoalbuminemia, electrolyte disturbances, fever, diarrhea, abnormal liver function, myelosuppression, allergy, vomiting, peripheral neuropathy, palpitations, thrombocytopenia, and neutropenia. The incidence of adverse events was comparable among the groups, with no statistically significant differences (Supplemental Table 6).

### Survival outcomes and mortality risk

**Figure 2.**
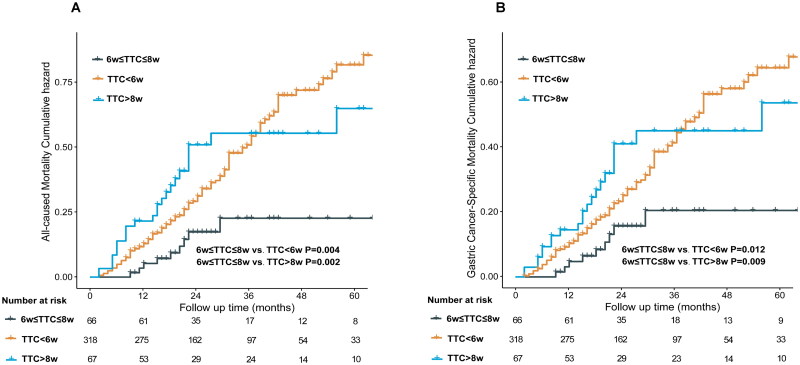
All-cause mortality (A) and gastric-cancer specific mortality (B) associated competitive risk assessed by TTC in this study population.

The median follow-up time for patients in this retrospective cohort study was 44.5 months. During this follow-up period, 150 deaths were observed, of which 139 were attributed to gastric cancer-related deaths. Cumulative competing risk curve analysis revealed that patients in the 6w ≤ TTC ≤ 8w group exhibited significantly lower 3-year ACM compared to the TTC < 6w group and the TTC > 8w group (3-year ACM: 19.7 *vs.* 37.2 *vs.* 39.7%, *p* = 0.009) (see Figure 2A). Regarding GCSM, patients in the 6w ≤ TTC ≤ 8w group showed significantly lower 3-year GCSM compared to the TTC < 6w group and the TTC > 8w group (3-year GCSM: 19.7 *vs.* 35.2 *vs.* 38.8%, *p* = 0.019) (see Figure 2B).

Further evaluation using the Cox regression model to assess independent prognostic factors associated with patient ACM, recurrence-free survival (RFS), and GCSM revealed that the American Society of Anesthesiologists (ASA) classification, ypTNM stage, tumor size, lymphovascular/neural invasion, TTC, and tumor regression grade (TRG) were prognostic factors for ACM in the univariate analysis. Upon inclusion of significant indicators (*p* < 0.05) from the univariate analysis into the multivariate analysis using the Cox regression model, it was found that ASA (ASA 3: HR: 2.391, 95% CI [1.329–4.299], *p* = 0.004), ypTNM stage (ypIII: HR: 2.747, 95% CI [1.409–5.358], *p* = 0.003), AC (AC (yes): HR: 0.641, 95% CI [0.455–0.904]), and TTC (TTC < 6w: HR: 3.102, 95% CI [1.559–6.171], *p* = 0.001; TTC > 8w: HR: 2.786, 95% CI [1.297–5.988], *p* = 0.009) were identified as independent prognostic risk factors for ACM (see Table 2). The same results were observed in the correlation analysis between GCSM and RFS (see Supplemental Tables 2 and 3).

**Table 2.** Univariate and multivariate analyses of factors associated with ACM.

Characteristic	Univariate analysis			Multivariate analysis		
	HR	95 CI	*p*-Value	HR	95 CI	*p*-Value
Age						
<65	REF					
≥65	0.988	0.714–1.369	0.944			
Sex						
Male	REF					
Female	1.106	0.780–1.569	0.572			
BMI						
<25 kg/m^2^	REF					
≥25 kg/m^2^	0.903	0.588–1.388	0.642			
ASA						
1	REF			REF		
2	1.273	0.769–2.105	0.348	1.377	0.827–2.292	0.219
3	2.642	1.488–4.693	**0.001**	2.391	1.329–4.299	**0.004**
ypStage						
ypCR/I	REF			REF		
II	1.371	0.717–2.622	0.340	1.061	0.527–2.134	0.869
III	3.587	2.016–6.383	**<0.001**	2.747	1.409–5.358	**0.003**
Tumor size						
<5 cm	REF			REF		
≥5 cm	1.496	1.086–2.061	**0.014**	1.120	0.797–1.572	0.514
Location						
Upper	REF					
Middle	1.052	0.671–1.650	0.824			
Lower	1.067	0.725–1.570	0.742			
Mixed	1.589	0927–2.727	0.092			
Lymphovascular invasion						
No	REF			REF		
Yes	1.564	1.113–2.159	**0.007**	1.107	0.769–1.594	0.585
Neural invasion						
No	REF			REF		
Yes	1.446	1.045–2.002	**0.026**	0.998	0.690–1.442	0.990
R status						
Negative	REF			REF		
Positive	1.617	1.017–2.571	**0.042**	1.147	0.712–1.849	0.573
AC						
<4 cycles	REF			REF		
≥4 cycles	0.713	0.515–0.985	**0.041**	0.641	0.455–0.904	**0.011**
TTC						
6w ≤ TTC ≤ 8w	REF			REF		
TTC < 6w	2.651	1.345–5.227	**0.005**	3.102	1.559–6.171	**0.001**
TTC > 8w	2.959	1.386–6.317	**0.005**	2.786	1.297–5.988	**0.009**
TRG grade						
0/1	REF			REF		
2/3	2.149	1.355–3.409	**0.001**	1.315	0.776–2.227	0.309
Complications						
No	REF					
Yes	1.307	0.919–1.859	0.137			

HR: hazard ratio; CI: confidence interval; BMI: body mass index; ASA: American Society of Anesthesiologists; ypCR/I, II, III: pathological nodal stage after neoadjuvant chemotherapy; tumor size: maximum diameter of the tumor; location: location of the tumor; TRG: tumor regression grade.

Bold values indicated that the *p*-value <0.05.

**Table 3.** Association of TTC with the risks of cancer-specific mortality and all-cause mortality among gastric cancer patients treated with neoadjuvant chemotherapy.

TTC (weeks)	Number of patients	Number of events	Model A^a^		Model B^b^		Model C^c^	
			HR (95% CI)	*p*	HR (95% CI)	*p*	HR (95% CI)	*p*
Gastric cancer-specific mortality								
6–8	66	9	Ref.		Ref.		Ref.	
<6	318	106	2.395 (1.204–4.766)	**.011**	2.591 (1.289–5.207)	**.008**	2.792 (1.382–5.641)	**.004**
>8	67	24	2.552 (1.171–5.563)	**.018**	2.341 (1.020–5.376)	**.024**	2.343 (1.060–5.180)	**.035**
All-cause mortality								
6–8	66	9	Ref.		Ref.		Ref.	
<6	318	115	2.736 (1.380–5.425)	**.004**	2.913 (1.457–5.824)	**.002**	3.102 (1.545–6.230)	**.001**
>8	67	26	3.004 (1.403–6.433)	**.005**	2.871 (1.326–6.215)	**.007**	2.719 (1.255–5.892)	**.011**

CI: confidence interval; HR: hazard ratio; TTC: time to initiation of adjuvant chemotherapy.

Bold values indicated that the *p*-value <0.05.

^a^ HRs were adjusted for age at diagnosis (as a continuous variable), sex, BMI (body mass index), ASA (American Society of Anesthesiologists).

^b^ HRs were additionally adjusted for pathological nodal stage after neoadjuvant chemotherapy (ypCR/I,II, III), tumor size (maximum diameter of the tumor), location (location of the tumor), lymphovascular invasion (positive or negative), neural invasion (positive or negative), and TRG (tumor regression grade).

^c^ HRs were additionally adjusted for R status(positive or negative), postoperative complication(yes or no), number of cycles of adjuvant chemotherapy (<4 or ≥4).

### TTC and adjusted models

Upon adjusting for demographic and preoperative characteristics (Model A), patients with TTC < 6w exhibited a 2.395-fold increased risk of GCSM (95% CI [1.204–4.766], *p* = 0.011) and a 2.736-fold increased risk of ACM (95% CI [1.380–5.425], *p* = 0.004) compared to those with 6w ≤ TTC ≤ 8w. Further adjustment for clinical factors and treatment modalities (Model C) strengthened and sustained the association between TTC < 6w and increased risk of GCSM and ACM relative to 6w ≤ TTC ≤ 8w (GCSM: HR: 2.792, 95% CI [1.382–5.641], *p* = 0.004; ACM: HR: 3.102, 95% CI [1.545–6.230], *p* = 0.001). Similarly, compared to the 6w ≤ TTC ≤ 8w group, patients with TTC > 8w had a 2.343-fold increased risk of GCSM (95% CI [1.060–5.180], *p* = 0.035) and a 2.719-fold increased risk of ACM (95% CI [1.255–5.892], *p* = 0.011) (see Table 3).

### TTC and mortality risks in subgroup analyses

Supplemental Table 4 illustrates the correlation between TTC and the risk of GCSM and ACM among patients with NLAGC after stratification into different subgroups based on tumor pathological stage, tumor size, lymphovascular invasion, neural invasion, and TRG grade. After adjusting for all confounding factors, no significant correlation between the risk of death and TTC was observed in patients with stage ypCR/I/II disease. However, among patients with stage III gastric cancer, those with TTC < 6w or >8w showed a 2.626-fold (95% CI [1.186–5.814], *p* = 0.017) and 2.875-fold (95% CI [1.139–7.252], *p* = 0.025) increased risk of GCSM, respectively, and a 2.908-fold (95% CI [1.323–6.391], *p* = 0.008) and 3.045-fold (95% CI [1.234–7.516], *p* = 0.016) increased risk of ACM, respectively. Similar results were also found in subgroups of patients with gastric cancer with tumor size > 5 cm, positive lymphovascular and neural invasion, and TRG 2/3 grade.

### Correlation of TTC with overall survival under different regimens and correlation of TTC with time to relapse

We conducted a stratified analysis based on different chemotherapy regimens to evaluate the impact of TTC on prognosis within each regimen group. The analysis showed that in the Paclitaxel regimens group, patients with a TTC of 6–8 weeks had significantly better 3-year overall survival compared to those with TTC < 6 weeks and TTC > 8 weeks (3-year OS: 96.6 *vs.* 79.5 *vs.* 64.1%; *p* = 0.026). Although similar results were not observed in the Platinum-based regimens and Other regimens groups, we found a trend toward better 3-year overall survival in the Platinum-based regimens group for patients with a TTC of 6–8 weeks compared to those with TTC < 6 weeks and TTC > 8 weeks (3-year OS: 79.9 *vs.* 57.6 *vs.* 59.9%; *p* = 0.109) (see Supplemental Figure 4).

**Figure 3.**
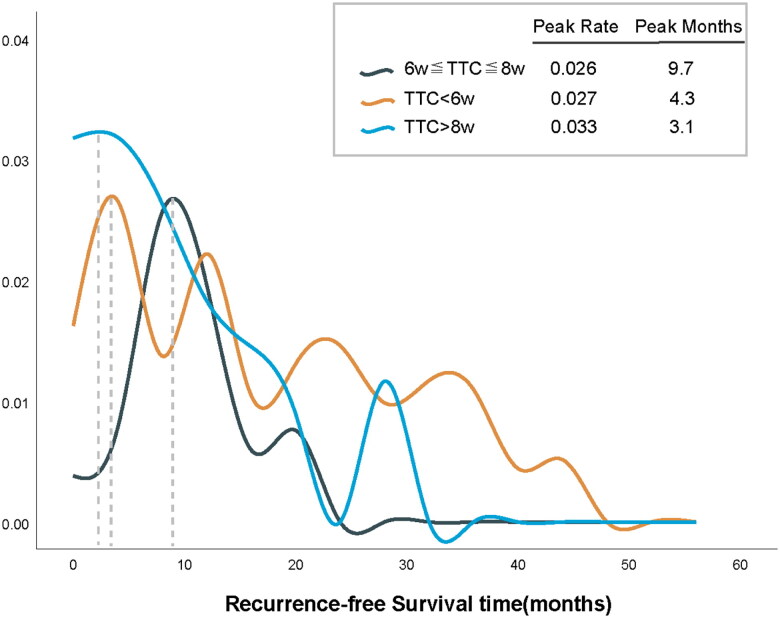
Whole-population comparison of time to relapse peaks stratified by TTC.

Simultaneously, in comparing the peak time of recurrence across different TTC stratifications within the entire population, we observed that the recurrence peak period in the 6w ≤ TTC ≤ 8w group was significantly prolonged compared to the TTC < 6w and TTC > 8w groups (Peak months: 9.7 *vs.* 4.3 *vs.* 3.1) (see Figure 3).

### Clinical implications of chemotherapy timing

Our study found that initiating chemotherapy within the 6–8 week window was associated with improved survival outcomes and delayed recurrence. This timeframe may serve as a reference for clinicians in determining the optimal timing for postoperative chemotherapy in gastric cancer patients, particularly those who have undergone neoadjuvant chemotherapy for locally advanced disease. These findings may contribute to the development of more personalized treatment strategies and potentially improve patient prognosis by reducing the risk of mortality.

## Discussion

Surgical intervention remains the primary approach for patients with LAGC, yet the 5-year survival rate for those solely undergoing surgery stands at a mere 20–30% [40]. Extensive prospective studies have underscored the clinical significance of perioperative chemotherapy, establishing it as the standard treatment for LAGC. In studies on other cancers, such as breast cancer [41,42] and ovarian cancer [43,44], it has been shown that initiating postoperative chemotherapy within an appropriate time window has a positive effect on patients receiving neoadjuvant therapy, helping to reduce recurrence and improve survival rates. However, with an increasing cohort of gastric cancer patients undergoing neoadjuvant therapy [4,5], the optimal timing for commencing postoperative adjuvant chemotherapy after neoadjuvant chemotherapy remains ambiguous regarding its impact on patient prognosis.

In this study, we identified a potentially applicable range of time for initiating postoperative adjuvant chemotherapy in patients with LAGC, namely within 6 to 8 weeks after surgery (6w ≤ TTC ≤ 8w). We found that patients who initiated postoperative chemotherapy within this range had significantly lower ACM and GCSM scores than those with TTC < 6w and TTC > 8w. After adjusting for all confounding factors, the TTC remained an independent prognostic factor for ACM and GCSM. To the best of our knowledge, this is the first study to explore the optimal timing for initiating postoperative maintenance chemotherapy in patients with locally advanced gastric cancer following neoadjuvant chemotherapy.

According to extant research, the initiation time of postoperative adjuvant chemotherapy significantly impacts the survival prognosis of various cancers, including LAGC [32,45]. This influence also extends to patients undergoing neoadjuvant therapy, with effects varying among different patient populations. Currently, published studies exhibit discrepancies in selecting the cutoff time for initiating postoperative chemotherapy. The management plan outlined in the Japanese Gastric Cancer Treatment Guidelines suggests that S-1 (adjuvant chemotherapy) should commence within 6 weeks post-surgery, following sufficient recovery from surgical intervention [3]. Both CRC and gastric cancer are malignant tumors of the digestive system. According to Dienstmann et al., an alternative cutoff point for TTC is reported to be 8 weeks post-CRC surgery for commencing adjuvant chemotherapy [46]. Due to both centers’ standard recommendation for patients to commence adjuvant chemotherapy within 6 weeks post-surgery, there was a higher proportion of patients in the TTC < 6w group (70.5%; 318/451) in the study. Our study revealed that patients who initiated chemotherapy within six weeks postoperatively exhibited significantly higher ACM and GCSM values than those within 6w ≤ TTC ≤ 8w. After adjusting for demographic and preoperative characteristics, patients with a TTC < 6w had a 2.395-fold increased risk of GCSM and a 2.736-fold increased risk of ACM. With further adjustments for tumor-related factors and treatment modalities, this relationship strengthened and remained significant.

The recovery of postoperative physiological function in patients with malignant gastric tumors necessitates time. Initiating postoperative adjuvant chemotherapy before a patient’s physiological function fully recovers may exacerbate harm to the patient [47]. Substantial evidence from RCTs confirms the efficacy of NACT in reducing the LAGC stage and improving patient prognosis [48]. However, NACT may elevate postoperative complications [49,50], and the recovery of physiological function after NACT may be inferior to that of patients who did not receive NACT. Therefore, we posit that a reasonable delay in postoperative chemotherapy is appropriate for patients undergoing NACT. Furthermore, in our study, we observed a 2.719-fold increase in the risk of ACM in patients with TTC > 8w compared to those with 6w ≤ TTC ≤ 8w (95% CI, 1.255–5.892; *p* = 0.011), along with an earlier recurrence peak (recurrence peak time: 3.1 *vs.* 9.7 months, respectively). This suggests that an excessive delay in initiating postoperative chemotherapy could have adverse prognostic implications for patients.

Previous studies, for instance, Carbognin et al. [51] used sensitivity analysis to evaluate the interaction between paclitaxel and docetaxel in terms of treatment efficacy and adverse effects, concluding that paclitaxel demonstrated a higher pathological complete response (pCR) rate and a lower incidence of severe neutropenia and febrile neutropenia, thereby strengthening the reliability of their findings. Similarly, Mueller et al. [52] applied sensitivity analysis to clarify the advantages of selectively using neoadjuvant chemoradiotherapy under different survival assumptions in locally advanced rectal cancer. Their study provided important clinical decision-making insights, showing that selective treatment strategies could balance efficacy while reducing costs. In this study, we conducted sensitivity analyses across different subgroups, including tumor pathological stage, tumor size, lymphovascular invasion, perineural invasion, and tumor regression grade (TRG), to evaluate the relationship between TTC and survival outcomes in LAGC patients (Supplemental Table 4). Among stage III gastric cancer patients, both TTC < 6 weeks and TTC > 8 weeks were associated with significantly higher risks of gastric cancer-specific mortality (GCSM) and all-cause mortality (ACM) compared to the standard 6–8 week window. Specifically: TTC < 6 weeks: GCSM risk increased by 2.626-fold (95% CI [1.186–5.814], *p* = 0.017); ACM risk increased by 2.908-fold (95% CI [1.323–6.391], *p* = 0.008). TTC > 8 weeks: GCSM risk increased by 2.875-fold (95% CI [1.139–7.252], *p* = 0.025); ACM risk increased by 3.045-fold (95% CI [1.234–7.516], *p* = 0.016). A similar trend was observed in patients with: Tumor size > 5 cm; Positive lymphovascular invasion and perineural invasion; TRG 2/3. These results suggest that sensitivity analysis further validates the impact of chemotherapy timing on survival outcomes in stage III gastric cancer patients and those with adverse tumor characteristics, thereby providing additional evidence for clinical decision-making.

Furthermore, we found that patients who initiated chemotherapy within 6–8 weeks had a lower risk of recurrence, whereas those who started chemotherapy earlier than 6 weeks or later than 8 weeks exhibited a relatively higher risk. This phenomenon may be associated with the following biological and clinical mechanisms: Firstly, the primary objective of postoperative adjuvant chemotherapy for gastric cancer is to mitigate the risk of tumor recurrence by eradicating residual malignant cells and inhibiting micrometastasis growth, thereby enhancing patient survival. The longer the interval between surgery and adjuvant chemotherapy, the greater the chance of micrometastasis amplification [11,12,53]. The rapid proliferation of residual cancer cells could render adjuvant chemotherapy beyond a certain time threshold minimally impactful on survival outcomes [54]. Animal model studies have also suggested that surgery may increase the number of circulating tumor cells, which could enhance metastatic growth and be associated with reduced angiogenesis and elevated oncogenic growth factors [13,14]. Secondly, Previous studies have indicated that gastric cancer patients require a certain period for physiological recovery after surgery. Initiating chemotherapy too early, before sufficient recovery, may impose an excessive burden on patients, leading to suboptimal treatment outcomes. In particular, early chemotherapy could excessively suppress the immune system, impairing immune recovery and negatively impacting long-term survival and recurrence [45]. However, not all patients voluntarily delay chemotherapy. Previous studies have indicated that advanced age, socioeconomic status, insurance coverage, and postoperative complications, influence chemotherapy timing [15,55,56]. Park et al. [22] reported that the most common reason for delayed chemotherapy beyond 8 weeks in gastric cancer patients was postoperative complications, while Datta et al. [57] found that most patients who experienced unplanned postoperative readmissions did not receive adjuvant treatment. These factors can affect subsequent treatment and indirectly impact patient prognosis. In this study, we adjusted for potential confounders using multivariate Cox regression analysis, including patient characteristics: age (≥65 *vs.* <65 years), sex (male *vs.* female), and BMI (≥25 *vs.* <25 kg/m^2^); perioperative status: ASA score (1/2/3), reflecting baseline health status and perioperative tolerance; tumor characteristics: ypStage (pathological stage), tumor size (≥5 *vs.* <5 cm), tumor location (upper/middle/lower/mixed), lymphovascular invasion, neural invasion, and R0/R1 resection status; and treatment-related factors: postoperative chemotherapy cycles (≥4 *vs.* <4 cycles), chemotherapy initiation time (TTC <6, 6–8, >8 weeks), and tumor regression grade (TRG 0/1 *vs.* 2/3). Through multivariate analysis, we eliminated these potential confounding factors and found that TTC remained an independent prognostic factor. Initiating chemotherapy within 6–8 weeks may provide an optimal time window that balances sufficient recovery with effective tumor suppression.

To further clarify the reasons for delayed chemotherapy, we compared the incidence of postoperative complications between the TTC > 8w and TTC ≤ 8w groups (see Supplemental Table 5). The results showed that the incidence of postoperative complications was higher in the TTC > 8w group compared to the TTC ≤ 8w group (15.3 *vs.* 14.9%), and the incidence of Clavien-Dindo grade ≥ II complications was also higher in the TTC > 8w group (6.5 *vs.* 11.9%). Therefore, the delay in TTC may be potentially associated with the occurrence of postoperative complications. Thus, avoiding excessive delays in initiating chemotherapy and selecting the appropriate time to commence postoperative chemotherapy is imperative.

In this study, we also found that the timing of postoperative chemotherapy initiation had a stronger impact on prognosis than R0 resection. However, when we compared the R1 resection rates and R0 resection rates among the three groups (TTC < 6w, 6w ≤ TTC ≤ 8w, and TTC > 8w), we found that the R1 resection rates were 10.1, 15.2, and 14.9%, respectively, which were significantly lower than the R0 resection rates in each group (89.9, 84.8, and 85.1%, respectively) (see Table 1). It is likely that the large difference in sample sizes between patients with R0 and R1 resections led to a relatively weaker association between R0 resection and postoperative prognosis, whereas the impact of the timing of postoperative chemotherapy initiation was more significant in the statistical analysis. In the multivariate analysis, we also found no significant differences in the efficacy of preoperative chemotherapy (TRG grade) among patients, but differences were observed in the timing of chemotherapy initiation. This may be because TRG primarily assesses tumor response to NACT, reflecting the effectiveness of preoperative chemotherapy. However, TRG does not directly involve postoperative treatment or control of tumor recurrence. A study by Kim et al. indicated that although patients with complete pathological response (TRG1a) are expected to have favorable survival outcomes, those with TRG1b had worse survival outcomes than patients with TRG2. Therefore, the association between pathological response and survival outcomes is not entirely consistent [58]. Similar results have been observed in other studies [35,59]. Thus, while TRG is a good predictor of response to NACT, other direct prognostic indicators, such as the timeliness of postoperative treatment (chemotherapy), may be more important for long-term survival [60].

Our study presents several limitations. Firstly, being a retrospective study, it is subject to selection bias. However, this study minimized bias as much as possible by adopting consecutive patient enrollment and conducting multivariable analysis. Secondly, there were relatively few cases within the 6 to 8-week timeframe after initiating postoperative chemotherapy among patients with LAGC who received NACT. However, these real-world clinical data analyses provide valuable insights into the optimal timing of postoperative chemotherapy across different cancer types. Thirdly, due to the retrospective nature of data collection in this study, we did not adequately collect and comprehensively record relevant data on genetic factors and socioeconomic variables. Further discussion on their relevance is needed in future research. Retrospective studies like ours remain crucial for identifying trends and generating hypotheses that guide future research. As the first study focusing on the optimal timing for postoperative chemotherapy initiation in LAGC patients receiving NACT, we hope it provides valuable reference for future study. Since this study is based on a Chinese patient cohort, the global applicability of our findings may be somewhat limited. Therefore, future research should aim to validate these results through large-scale, multicenter, and prospective studies to enhance the generalizability of the conclusions.

## Conclusion

Patients with NLAGC and a TTC between 6 and 8 weeks may have lower GCSM and ACM compared to those with TTC < 6 weeks or more than 8 weeks. Efforts should be made to initiate postoperative adjuvant chemotherapy at appropriate times, especially for high-risk patients with ypTNM stage III, tumor size of 5 cm or larger, positive lymphovascular/neural invasion, and TRG 2/3 grades.

This study has significant implications for clinical practice, highlighting that the timing of postoperative adjuvant chemotherapy should be considered a critical factor in treatment decision-making. The findings provide preliminary evidence to support potential revisions to current treatment guidelines for gastric cancer patients receiving neoadjuvant chemotherapy. Since this study is retrospective, large-scale, multicenter, prospective clinical trials are needed to further validate the impact of different chemotherapy timing strategies on survival outcomes in stage III gastric cancer patients and those with specific adverse tumor characteristics.

## Supplementary Material

IANN-2024-5913.R1-Supplementary Table clean copy.docx

## Data Availability

The dataset generated for this current study is not publicly available due to additional research questions to be answered but is available from the corresponding author on reasonable request.
