# Supplementary material for: Timing of postoperative chemotherapy and prognosis in neoadjuvant-treated gastric cancer patients: a multicenter real-world cohort study
Source: Ann Med. 2025 May 7;57(1):2500690. doi: 10.1080/07853890.2025.2500690 (PMC12064125; doi:10.1080/07853890.2025.2500690)

Supplementary Information

Supplemental Table 1: Baseline Characteristics of Patients by Chemotherapy Regimen and Timing of Chemotherapy Initiation

Supplemental Table 2：Univariate and multivariate analyses of factors associated with RFS

Supplemental Table 3: Univariate and multivariate analyses of factors associated with GCSM

Supplemental Table 4: Correlation of TTC with ACM and GCSM in each subgroup of tumor-related data populations

Supplemental Table 5: Comparison of postoperative complications between TTC≤8w and TTC >8w

Supplemental Table 6 Incidence of Chemotherapy-related Adverse Reactions Among Different TTC Groups.

Supplemental Figure 1: A. Distribution of chemotherapy regimens in the entire cohort. B. Distribution of chemotherapy regimens by TTC

Supplemental Figure 2：Definition: Time to chemotherapy:(TTC): The time interval between the date of surgery and the initiation of postoperative adjuvant chemotherapy

Supplemental Figure 3. A. Distribution of neoadjuvant chemotherapy cycles in the entire cohort. B. Distribution of postoperative adjuvant chemotherapy cycles in the entire cohort

Supplemental Figure 4. The Kaplan-Meier analysis of postoperative overall survival in different chemotherapy regimen subgroups based on TTC. A: in the Platinum-based regimens population; B: in the Paclitaxel regimens population; C: in the others regimens population

Supplemental Figure 5. Difference in chemotherapy regimens between different time periods.

Supplementary Table 1 Baseline Characteristics of Patients by Chemotherapy Regimen and Timing of Chemotherapy Initiation

| Variate | platinum-based regimens | | | | paclitaxel regimens | | | | Other regimens | | | |
| --- | --- | --- | --- | --- | --- | --- | --- | --- | --- | --- | --- | --- |
|  | TTC<6  n = 121 | 6≤TTC≤8  n= 26 | TTC>8  n = 33 | P | TTC<6  n = 129 | 6≤TTC≤8  n = 29 | TTC>8  n = 22 | P | TTC<6  n = 68 | 6≤TTC≤8,  n = 11 | TTC>8  n = 12 | P |
| Age,n(%) |  |  |  | 0.295 |  |  |  | 0.301 |  |  |  | 0.04 |
| <65 | 75 (62.0%) | 12 (46.2%) | 18 (54.5%) |  | 65 (50.4%) | 11 (37.9%) | 13 (59.1%) |  | 50 (73.5%) | 4 (36.4%) | 7 (58.3%) |  |
| ≥65 | 46 (38.0%) | 14 (53.8%) | 15 (45.5%) |  | 64 (49.6%) | 18 (62.1%) | 9 (40.9%) |  | 18 (26.5%) | 7 (63.6%) | 5 (41.7%) |  |
| Sex,n(%) |  |  |  | 0.215 |  |  |  | 0.189 |  |  |  | 0.693 |
| male | 84 (69.4%) | 19 (73.1%) | 18 (54.5%) |  | 104 (80.6%) | 22 (75.9%) | 14 (63.6%) |  | 46 (67.6%) | 9 (81.8%) | 9 (75.0%) |  |
| female | 37 (30.6%) | 7 (26.9%) | 15 (45.5%) |  | 25 (19.4%) | 7 (24.1%) | 8 (36.4%) |  | 22 (32.4%) | 2 (18.2%) | 3 (25.0%) |  |
| ASA,n(%) |  |  |  | 0.386 |  |  |  | 0.076 |  |  |  | 0.932 |
| 1 | 22 (18.2%) | 3 (11.5%) | 2 (6.1%) |  | 14 (10.9%) | 3 (10.3%) | 6 (27.3%) |  | 10 (14.7%) | 1 (9.1%) | 2 (16.7%) |  |
| 2 | 84 (69.4%) | 18 (69.2%) | 27 (81.8%) |  | 93 (72.1%) | 23 (79.3%) | 10 (45.5%) |  | 50 (73.5%) | 9 (81.8%) | 8 (66.7%) |  |
| 3 | 15 (12.4%) | 5 (19.2%) | 4 (12.1%) |  | 22 (17.1%) | 3 (10.3%) | 6 (27.3%) |  | 8 (11.8%) | 1 (9.1%) | 2 (16.7%) |  |
| ypStage,n(%) |  |  |  | 0.882 |  |  |  | 0.288 |  |  |  | 0.296 |
| ypCR/Ⅰ | 19 (15.7%) | 5 (19.2%) | 6 (18.2%) |  | 32 (24.8%) | 9 (31.0%) | 6 (27.3%) |  | 9 (13.2%) | 3 (27.3%) | 0 (0.0%) |  |
| Ⅱ | 38 (31.4%) | 6 (23.1%) | 8 (24.2%) |  | 48 (37.2%) | 12 (41.4%) | 4 (18.2%) |  | 20 (29.4%) | 2 (18.2%) | 2 (16.7%) |  |
| Ⅲ | 64 (52.9%) | 15 (57.7%) | 19 (57.6%) |  | 49 (38.0%) | 8 (27.6%) | 12 (54.5%) |  | 39 (57.4%) | 6 (54.5%) | 10 (83.3%) |  |
| Tumor size(cm) | 4.00  (3.00,6.00) | 5.00  (3.25, 6.00) | 4.50  (2.50, 5.50) | 0.399 | 4.00  (3.00, 5.50) | 4.10  (3.05, 5.50) | 3.50  (2.90, 4.50) | 0.444 | 4.65 (  3.15, 7.00) | 5.00  (2.90, 6.50) | 4.75  (3.50,7.50) | 0.701 |
| Lymphovascular invasion,  n(%) |  |  |  | 0.673 |  |  |  | 0.621 |  |  |  | 0.880 |
| no | 50 (41.3%) | 13 (50.0%) | 13 (39.4%) |  | 48 (37.2%) | 13 (44.8%) | 10 (45.5%) |  | 29 (42.6%) | 5 (45.5%) | 4 (33.3%) |  |
| yes | 71 (58.7%) | 13 (50.0%) | 20 (60.6%) |  | 81 (62.8%) | 16 (55.2%) | 12 (54.5%) |  | 39 (57.4%) | 6 (54.5%) | 8 (66.7%) |  |
| Neural invasion,n(%) |  |  |  | 0.618 |  |  |  | 0.033 |  |  |  | 0.139 |
| no | 64 (52.9%) | 11 (42.3%) | 17 (51.5%) |  | 62 (48.1%) | 17 (58.6%) | 5 (22.7%) |  | 34 (50.0%) | 9 (81.8%) | 6 (50.0%) |  |
| yes | 57 (47.1%) | 15 (57.7%) | 16 (48.5%) |  | 67 (51.9%) | 12 (41.4%) | 17 (77.3%) |  | 34 (50.0%) | 2 (18.2%) | 6 (50.0%) |  |
| TRGgrade  ,n(%) |  |  |  | 0.796 |  |  |  | 0.288 |  |  |  | 0.056 |
| 0/1 | 23 (19.0%) | 5 (19.2%) | 8 (24.2%) |  | 43 (33.3%) | 11 (37.9%) | 4 (18.2%) |  | 10 (14.7%) | 5 (45.5%) | 3 (25.0%) |  |
| 2/3 | 98 (81.0%) | 21 (80.8%) | 25 (75.8%) |  | 86 (66.7%) | 18 (62.1%) | 18 (81.8%) |  | 58 (85.3%) | 6 (54.5%) | 9 (75.0%) |  |
| AC,n(%) |  |  |  | 0.563 |  |  |  | 0.132 |  |  |  | 0.063 |
| <4 cycles | 51 (42.1%) | 9 (34.6%) | 16 (48.5%) |  | 47 (36.4%) | 12 (41.4%) | 13 (59.1%) |  | 15 (22.1%) | 3 (27.3%) | 7 (58.3%) |  |
| ≥4 cycles | 70 (57.9%) | 17 (65.4%) | 17 (51.5%) |  | 82 (63.6%) | 17 (58.6%) | 9 (40.9%) |  | 53 (77.9%) | 8 (72.7%) | 5 (41.7%) |  |
| LOS, median(IQR) | 9.0  (7.0, 11.0) | 9.0  (7.3, 12.0) | 10.0  (7.0, 13.0) | 0.118 | 7.00  (7.00, 9.00) | 8.00  (7.00, 12.00) | 7.00  (7.00, 9.00) | 0.325 | 8.0  (7.0, 11.0) | 11.0  (7.5, 16.0) | 7.0  (7.0, 8.8) | 0.214 |
| Duration of Operation, median(IQR) | 189  (155, 220) | 178  (151, 200) | 180  (145, 236) | 0.42 | 190  (165, 231) | 200  (180, 210) | 180  (161, 216) | 0.478 | 192  (166, 235) | 180  (165, 260) | 215  (157, 225) | 0.997 |
| Blood loss, median(IQR) | 50  (35, 80) | 50  (30, 50) | 50  (35, 80) | 0.452 | 30  (30, 50) | 35  (30, 50) | 40  (30, 50) | 0.682 | 50  (30, 100) | 50  (43, 93) | 50  (45, 100) | 0.808 |

Bold values indicated that the P value < 0.05.

Abbreviation: BMI (Body Mass Index),ASA (American Society of Anesthesiologists）,pathological nodal stage after neoadjuvant chemotherapy (ypCR/I,II, III), tumor size(maximum diameter of the tumor), location(location of the tumor)and TRG(tumor regression grade), AC(adjuvant chemotherapy), LOS(length of stay)

Supplementary Table 2:Univariate and multivariate analyses of factors associated with RFS

| Characteristic | Univariate analysis | | | Multivariate analysis | | |
| --- | --- | --- | --- | --- | --- | --- |
|  | HR^1^ | 95 CI^1^ | p-value | HR^1^ | 95 CI^1^ | p-value |
| Age |  |  |  |  |  |  |
| <65 | REF | |  |  |  |  |
| ≥65 | 0.898 | 0.656-1.227 | 0.499 |  |  |  |
| Sex |  |  |  |  |  |  |
| male | REF | |  |  |  |  |
| female | 1.078 | 0.770-1.510 | 0.662 |  |  |  |
| BMI |  |  |  |  |  |  |
| <25kg/m2 | REF | |  |  |  |  |
| ≥25kg/m2 | 0.851 | 0.563-1.286 | 0.442 |  |  |  |
| ASA |  |  |  |  |  |  |
| 1 | REF | |  | REF | |  |
| 2 | 1.064 | 0.672-1.683 | 0.792 | 1.264 | 0.796-2.0006 | 0.321 |
| 3 | 1.934 | 1.126- 3.321 | 0.017 | 2.035 | 1.179-3.514 | 0.011 |
| ypStage |  |  |  |  |  |  |
| ypCR/Ⅰ | REF | |  | REF | |  |
| Ⅱ | 1.449 | 0.776-2.708 | 0.245 | 1.091 | 0.552-2.157 | 0.801 |
| Ⅲ | 4.112 | 2.361-7.162 | <0.001 | 2.727 | 1.437-5.175 | 0.002 |
| Tumor size |  |  |  |  |  |  |
| <5cm | REF | |  | REF | |  |
| ≥5cm | 1.699 | 1.250-2.311 | 0.001 | 1.297 | 0.938-1.792 | 0.116 |
| Location |  |  |  |  |  |  |
| upper | REF | |  |  |  |  |
| middle | 1.124 | 0.733-1.722 | 0.592 |  |  |  |
| lower | 1.060 | 0.732-1.535 | 0.756 |  |  |  |
| mixed | 1.485 | 0.871-2.531 | 0.146 |  |  |  |
| **Lymphovascular invasion** |  |  |  |  |  |  |
| no | REF | |  | REF | |  |
| yes | 1.744 | 1.282-2.372 | <0.001 | 1.125 | 0.811-1.562 | 0.480 |
| Neural invasion |  |  |  |  |  |  |
| no | REF | |  |  |  |  |
| yes | 1.322 | 0.970-1.802 | 0.077 |  |  |  |
| R status |  |  |  |  |  |  |
| negative | REF | |  | REF | |  |
| Positive | 1.979 | 1.307-2.996 | 0.001 | 1.544 | 1.006-2.370 | 0.047 |
| **AC** |  |  |  |  |  |  |
| **<4 cycles** | REF | |  |  |  |  |
| **≥4 cycles** | 0.866 | 0.632-1.186 | 0.369 |  |  |  |
| TTC |  |  |  |  |  |  |
| 6w≤TTC≤8w | REF | |  | REF | |  |
| TTC<6w | 1.767 | 1.033-3.022 | 0.038 | 1.991 | 1.156-3.430 | 0.013 |
| TTC>8w | 2.000 | 1.064-3.761 | 0.031 | 2.026 | 1.067-3.845 | 0.031 |
| TRG grade |  |  |  |  |  |  |
| 0/1 | REF | |  | REF | |  |
| 2/3 | 2.308 | 1.485- 3.588 | <0.001 | 1.367 | 0.827-2.262 | 0.223 |
| Complications |  |  |  |  |  |  |
| no | REF | |  |  |  |  |
| yes | 1.391 | 0.989-1.958 | 0.058 |  |  |  |
| ^1^HR =Hazard Ratio, CI = Confidence Interval  Bold values indicated that the P value < 0.05  Abbreviation: BMI (Body Mass Index),ASA (American Society of Anesthesiologists）,pathological nodal stage after neoadjuvant chemotherapy (ypCR/I,II, III), tumor size(maximum diameter of the tumor), location(location of the tumor)and TRG(tumor regression grade) | | | | | | |

Supplementary Table 3：Univariate and multivariate analyses of factors associated with GCSM

| Characteristic | Univariate analysis | | | Multivariate analysis | | |
| --- | --- | --- | --- | --- | --- | --- |
|  | HR^1^ | 95% CI^1^ | p-value | HR^1^ | 95% CI^1^ | p-value |
| Age |  |  |  |  |  |  |
| <65 | REF | |  |  |  |  |
| ≥65 | 0.931 | 0.658-1.318 | 0.687 |  |  |  |
| Sex |  |  |  |  |  |  |
| male | REF | |  |  |  |  |
| female | 1.067 | 0.734-1.550 | 0.734 |  |  |  |
| BMI |  |  |  |  |  |  |
| <25kg/m2 | REF | |  |  |  |  |
| ≥25kg/m2 | 0.902 | 0.571-1.425 | 0.657 |  |  |  |
| ASA |  |  |  |  |  |  |
| 1 | REF | |  | REF | |  |
| 2 | 1.330 | 0.768-2.303 | 0.309 | 1.424 | 0.820-2.475 | 0.209 |
| 3 | 2.968 | 1.602-5.496 | 0.001 | 2.579 | 1.376-4.834 | 0.003 |
| ypStage |  |  |  |  |  |  |
| ypCR/Ⅰ | REF | |  | REF | |  |
| Ⅱ | 1.673 | 0.815-3.433 | 0.161 | 1.332 | 0.617-2.880 | 0.465 |
| Ⅲ | 4.128 | 2.150-7.925 | <0.001 | 3.148 | 1.500-6.606 | 0.002 |
| Tumor size |  |  |  |  |  |  |
| <5cm | REF | |  | REF | |  |
| ≥5cm | 1.608 | 1.144-2.259 | 0.006 | 1.201 | 0.838-1.721 | 0.318 |
| Location |  |  |  |  |  |  |
| upper | REF | |  |  |  |  |
| middle | 1.219 | 0.762-1.951 | 0.408 |  |  |  |
| lower | 1.150 | 0.759-1.742 | 0.511 |  |  |  |
| mixed | 1.669 | 0.932-2.988 | 0.085 |  |  |  |
| **Lymphovascular invasion** |  |  |  |  |  |  |
| no | REF | |  | REF | |  |
| yes | 1.655 | 1.176-2.329 | 0.004 | 1.166 | 0.808-1.684 | 0.412 |
| Neural invasion |  |  |  |  |  |  |
| no | REF | |  |  |  |  |
| yes | 1.357 | 0.962-1.914 | 0.082 |  |  |  |
| R status |  |  |  |  |  |  |
| negative | REF | |  | REF | |  |
| Positive | 1.772 | 1.098-2.859 | 0.019 | 1.270 | 0.776-2.078 | 0.342 |
| **AC** |  |  |  |  |  |  |
| **<4 cycles** | REF | |  | REF | |  |
| **≥4 cycles** | 0.703 | 0.499-0.992 | 0.045 | 0.631 | 0.439-0.909 | 0.013 |
| TTC |  |  |  |  |  |  |
| 6w≤TTC≤8w | REF | |  | REF | |  |
| TTC<6w | 2.345 | 1.186-4.638 | 0.014 | 2.804 | 1.405-5.594 | 0.003 |
| TTC>8w | 2.527 | 1.163-5.490 | 0.019 | 2.415 | 1.105-5.278 | 0.027 |
| TRG grade |  |  |  |  |  |  |
| 0/1 | REF | |  | REF | |  |
| 2/3 | 2.111 | 1.299- 3.432 | 0.003 | 1.178 | 0.682-2.035 | 0.558 |
| Complications |  |  |  |  |  |  |
| no | REF | |  |  |  |  |
| yes | 1.313 | 0.903-1.909 | 0.153 |  |  |  |
| ^1^HR =Hazard Ratio, CI = Confidence Interval  Bold values indicated that the P value < 0.05  Abbreviation: BMI (Body Mass Index),ASA (American Society of Anesthesiologists）,pathological nodal stage after neoadjuvant chemotherapy (ypCR/I,II, III), tumor size(maximum diameter of the tumor), location(location of the tumor)and TRG(tumor regression grade) | | | | | | |

Supplementary Table 4:Correlation of TTC with ACM and GCSM in each subgroup of tumor-related data populations

| Gastric-cancer specific mortality | | All-cause mortality | | |
| --- | --- | --- | --- | --- |
| TTC weeks | HR(95%CI)^a^ | *P*-value | HR(95%CI)^a^ | *P*-value |
| ypStage |  |  |  |  |
| ypCR/Ⅰ |  |  |  |  |
| 6-8 | Ref. |  | Ref. |  |
| <6 | 1.685(0.206-13.796) | 0.627 | 2.146(0.270-17.043) | 0.470 |
| >8 | 2.977(0.268-33.097) | 0.375 | 4.457(0.461-43.113) | 0.197 |
| Ⅱ |  |  |  |  |
| 6-8 | Ref. |  | Ref. |  |
| <6 | 4.213(0.570-31.138) | 0.159 | 4.373(0.593-32.269) | 0.148 |
| >8 | 3.317(0.344-31.937) | 0.299 | 4.379(0.488-39.259) | 0.187 |
| Ⅲ |  |  |  |  |
| 6-8 | Ref. |  | Ref. |  |
| <6 | 2.626(1.186-5.814) | 0.017 | 2.908(1.323-6.391) | 0.008 |
| >8 | 2.875(1.139-7.252) | 0.025 | 3.045(1.234-7.516) | 0.016 |
| Tumor size  (≥5cm) |  |  |  |  |
| 6-8 | Ref. |  | Ref. |  |
| <6 | 3.151(1.187-8.361) | 0.021 | 2.448(1.027-5.832) | 0.043 |
| >8 | 4.197(1.316-13.380) | 0.015 | 3.023(1.040-8.785) | 0.042 |
| Lymphovascular invasion  (positive) |  |  |  |  |
| 6-8 | Ref. |  | Ref. |  |
| <6 | 5.472(1.951-15.343) | 0.001 | 6.420(2.322-17.750) | <0.001 |
| >8 | 3.389(1.012-11.349) | 0.048 | 3.938(1.218-12.734) | 0.022 |
| Neural invasion  (postive) |  |  |  |  |
| 6-8 | Ref. |  | Ref. |  |
| <6 | 4.372(1.356-14.089) | 0.013 | 5.042(1.572-16.168) | 0.007 |
| >8 | 4.410(1.206-16.131) | 0.025 | 5.176(1.452-18.452) | 0.011 |
| TRG grade |  |  |  |  |
| 0/1 |  |  |  |  |
| 6-8 | Ref. |  | Ref. |  |
| <6 | 1.998(0.351-11.364) | 0.435 | 2.123(0.384-11.732) | 0.388 |
| >8 | 1.846(0.135-25.292) | 0.646 | 2.666(0.279-25.508) | 0.395 |
| 2/3 |  |  |  |  |
| 6-8 | Ref. |  | Ref. |  |
| <6 | 2.798(1.286-6.087) | 0.009 | 3.118(1.438-6.760) | 0.004 |
| >8 | 2.470(1.008-6.056) | 0.048 | 2.677(1.109-6.460) | 0.029 |
| Abbreviations: CI, confidence interval; HR, hazard ratio; TTR, time to initiation of adjuvant radiotherapy.  Bold values indicated that the P value < 0.05  ^a^HRs were adjusted for age at diagnosis (as a continuous variable), sex, BMI (Body Mass Index),ASA (American Society of Anesthesiologists）,pathological nodal stage after neoadjuvant chemotherapy (ypCR/I,II, III), tumor size(maximum diameter of the tumor), location(location of the tumor), Lymphovascular invasion(positive or negative), Neural invasion(positive or negative) and TRG(tumor regression grade),postoperative complication( yes or no).Note: Bold values indicated that the P< 0.05 | | | | |

Supplementary Table 5: Comparison of postoperative complications between TTC≤ 8w and TTC> 8w

| Variates | TTC≤8w  N=384 | TTC>8w  N=67 | P-value |
| --- | --- | --- | --- |
| Postoperative complications,n(%) |  |  | 0.368 |
| no | 300(78.1) | 49(73.1) |  |
| yes | 84(21.9) | 18 (26.9) |  |
| Clavien-Dindo grade |  |  | 0.227 |
| I | 59(15.3) | 10(14.9) |  |
| ≥ II | 25(6.5) | 8 (11.9) |  |

Supplemental Table 6: Incidence of Chemotherapy-related Adverse Reactions Among Different TTC Groups

| Variables | TTC<6  n = 318(%) | 6≤TTC≤8  n=66(%) | TTC>8  n=67(%) | P-value^a^ |
| --- | --- | --- | --- | --- |
|  |  |  |  |  |
| Chemotherapy-related Adverse Reactions, n(%) |  |  |  | 0.978 |
| Leukopenia | 0 | 0 | 2 (3.0) |  |
| Hypoproteinemia | 1 (0.30) | 0 | 3 (4.5) |  |
| Electrolyte imbalance | 0 | 1 (1.5) | 2 (3.0) |  |
| Fever | 0 | 0 | 2 (3.0) |  |
| Diarrhea | 1 (0.30) | 0 | 3 (4.5) |  |
| Abnormal liver function | 4 (1.2) | 2 (3.0) | 7 (10.5) |  |
| Myelosuppression | 3 (0.9) | 3 (4.5) | 5 (7.5) |  |
| Allergy | 0 | 0 | 3 (4.5) |  |
| Vomiting | 2 (0.6) | 2 (3.0) | 2 (3.0) |  |
| Peripheral neuropathy | 0 | 0 | 2 (3.0) |  |
| Palpitations | 0 | 1 (3.0) | 2 (3.0) |  |
| Thrombocytopenia | 0 | 1 (1.5) | 3 (4.5) |  |
| Neutropenia | 1 (0.3) | 1 (1.5) | 2 (3.0) |  |
| a: Fisher exact | | | | |

Supplementary Figure 1. A. Distribution of chemotherapy regimens in the entire cohort. B. Distribution of chemotherapy regimens by TTC.

A B


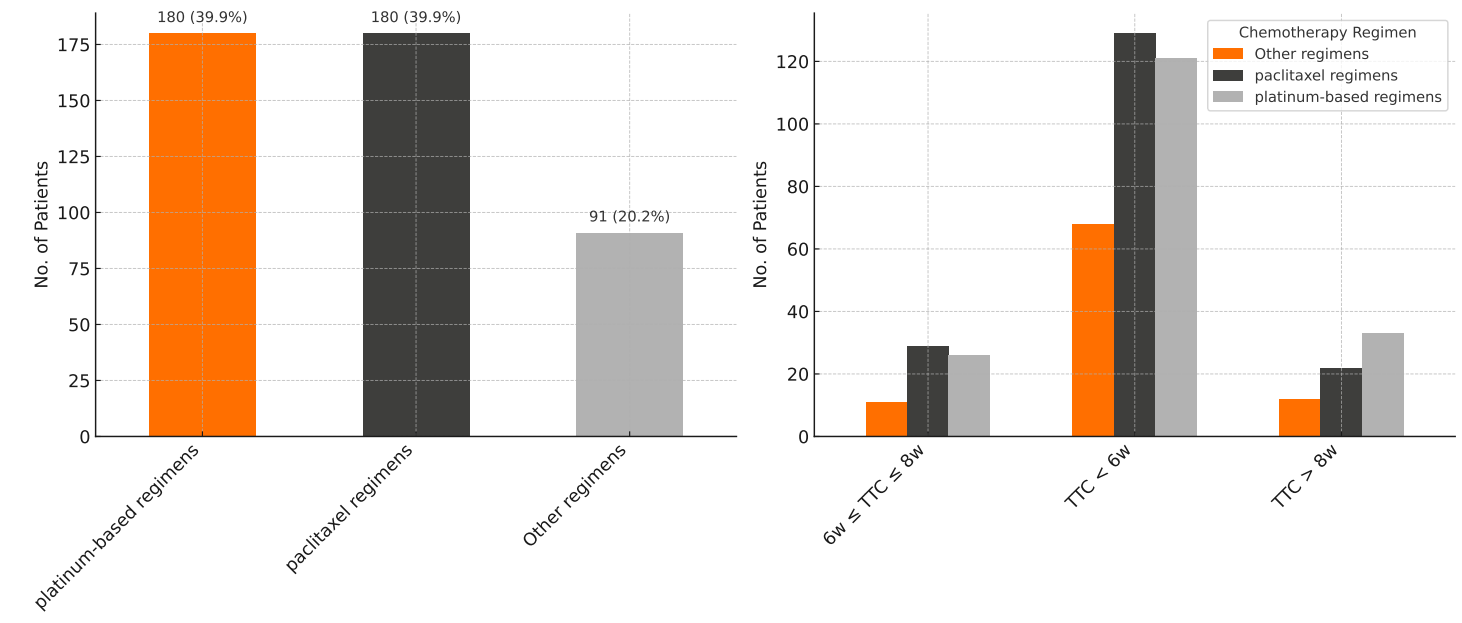


Supplementary Figure 2: Definition: Time to chemotherapy:(TTC): The time interval between the date of surgery and the initiation of postoperative adjuvant chemotherapy.


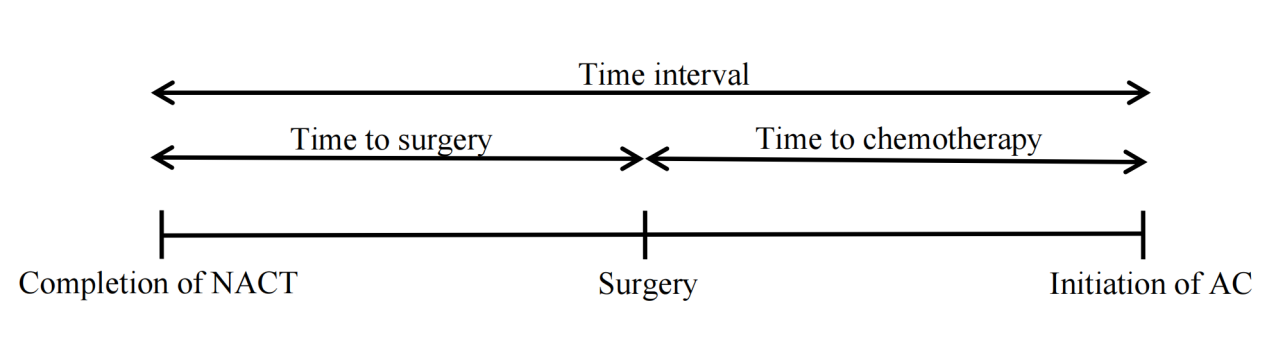


Supplementary Figure 3. A. Distribution of neoadjuvant chemotherapy cycles in the entire cohort. B. Distribution of postoperative adjuvant chemotherapy cycles in the entire cohort.


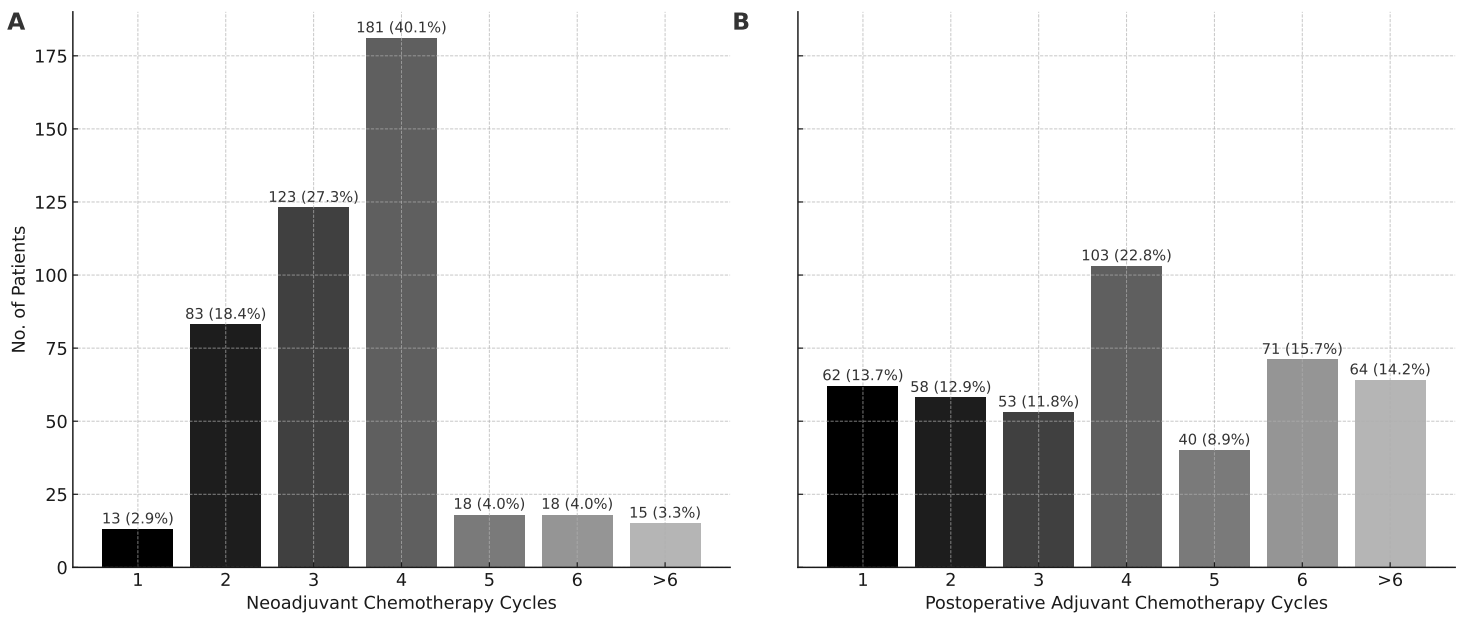


Supplementary
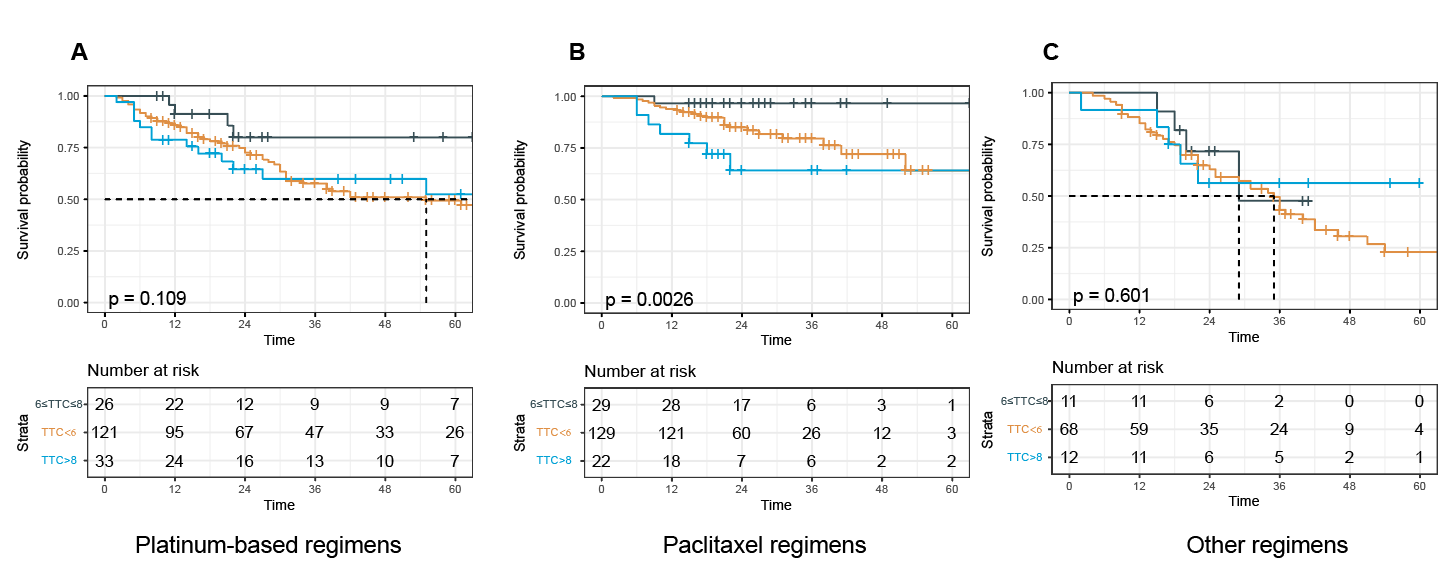
Figure 4. The Kaplan-Meier analysis of postoperative overall survival in different chemotherapy regimen subgroups based on TTC. A: in the Platinum-based regimens population; B: in the Paclitaxel regimens population; C: in the others regimens population.

Supplemental Figure 5. Difference in chemotherapy regimens between different time periods. Timeframe: 2010–2013: The treatment of locally advanced gastric cancer (LAGC) primarily relied on traditional chemotherapy regimens such as ECF (epirubicin, cisplatin, and fluorouracil) and XELOX (oxaliplatin and capecitabine).2014–2017: With the gradual updates to neoadjuvant chemotherapy standards, some regions began adopting more advanced regimens, including FOLFOX (fluorouracil, leucovorin, and oxaliplatin) and FLOT (docetaxel, oxaliplatin, leucovorin, and fluorouracil). 2018–2022: Neoadjuvant chemotherapy progressively shifted toward personalized treatment approaches.


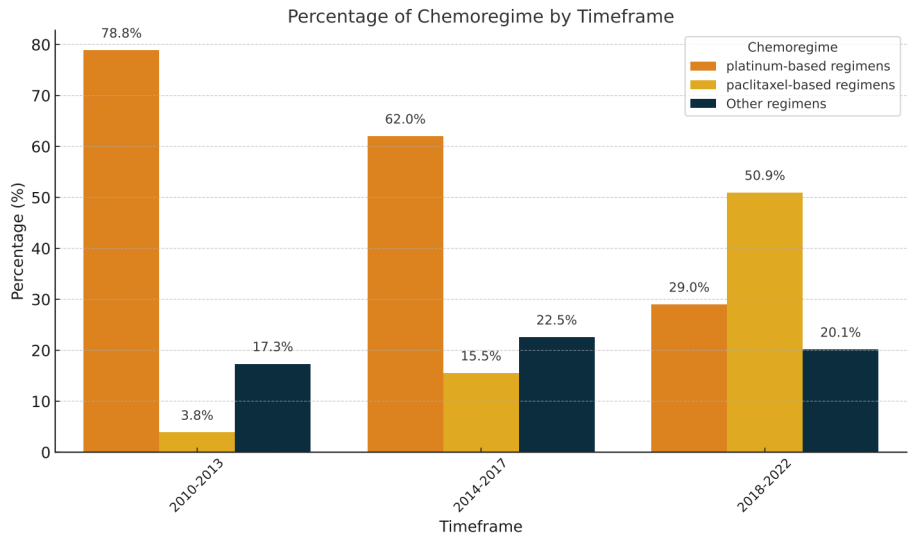

Supplement: IANN-2024-5913.R1-Supplementary Table clean copy.docx [file IANN_A_2500690_SM5827.docx]
